# Retraction Note: Long noncoding RNA CASC2 inhibits ox-LDL-mediated vascular smooth muscle cells proliferation and migration via the regulation of miR-532-3p/PAPD5

**DOI:** 10.1186/s10020-024-00966-w

**Published:** 2024-10-28

**Authors:** Chenjing Wang, Jin Zhao, Xiaodong Nan, Zhong Guo, Shuangsheng Huang, Xiaokun Wang, Feng Sun, Shijie Ma

**Affiliations:** 1 School of Basic Medical Sciences, Northwest Minzu University Health Science Center, No. 1, XibeiXincun Chengguan District, Lanzhou City, Gansu Province 730030 People’s Republic of China; 2 Department of intensive care unit, Gansu Provincial Corps Hospital of Chinese People’s Armed Police Force, Lanzhou City, Gansu Province 730050 People’s Republic of China

**Retraction Note: Molecular Medicine (2020) 26:74**

10.1186/s10020-020-00200-3

The Editor in Chief has retracted this article. After publication, concerns were raised regarding panel CASC2 in Fig. 4C. It appears to overlap with panel pcLINC00472 in Fig. 3c in [1] and panel miR-18a inhibitors in Fig. 3F in [2]. All three articles were in submission at the same time and were written by different author groups. None of the authors agrees to this retraction.
